# The correction of the urban-rural wealth Gini coefficient of China

**DOI:** 10.1371/journal.pone.0346669

**Published:** 2026-04-09

**Authors:** Pingsheng Dai, Zhaohui Jin

**Affiliations:** School of Finance and Economics, Jimei University, Xiamen, Fujian, China; Universidad de Chile, CHILE

## Abstract

Due to the lack of top wealth records in household surveys, many scholars believe that China’s wealth Gini coefficient is seriously underestimated. Based on data from CFPS (China Household Panel Survey), this paper corrects the urban-rural wealth Gini coefficient of China in combination with the Hurun Rich List. The study finds that real estate accounts for more than 75% of total assets, and financial assets account for less than 20% of total assets. Housing inequality contributes more than 75% to wealth inequality. The urban-rural decomposition of the wealth Gini coefficient shows that the contribution rate of urban-rural wealth inequality to the total wealth Gini coefficient is more than 50%. After the correction based on data from the Rich List, the urban wealth Gini coefficient of China is at the level of 0.70, and the urban-rural wealth Gini coefficient of China shows an inverted U-shaped change.

## 1. Introduction

The Chinese government’s goal of common prosperity is highly consistent with the shared prosperity actively advocated by the World Bank. The housing system reform implemented in China in 1994 and the traditional dual household registration system have contributed to the rapid widening of the wealth gap between urban and rural residents. According to estimates from the World Inequality Database (WID), China’s wealth Gini coefficient has fluctuated around 0.75 in recent years, making it one of the countries with the highest wealth inequality in the world.

Narrowing the urban-rural wealth gap is the key to achieving China’s development goal of common prosperity, for the following main reasons: First, the urban-rural wealth gap stems from imperfections in China’s social and economic systems. (1) For more than half a century, China has implemented a household registration system known as hukou, which restricts population mobility between urban and rural areas [1,2]. To accelerate industrialization, the government suppressed the prices of agricultural products and other raw materials from rural areas while raising the prices of industrial goods from urban areas. This policy was designed to increase the profits of state-owned enterprises and promote industrial development, gradually concentrating wealth in urban regions [3]. (2) Before the 1978 economic reforms, the government implemented a planned economy that almost completely eliminated private property, with land under state ownership. Rural residents retained private housing, whereas urban residents lived in public housing. Starting in 1988, the government launched housing reforms in urban areas, selling or renting public housing to urban residents [4]. The commercialization of housing enabled urban residents to acquire private property. Meanwhile, the government generated revenue through the transfer of state-owned land use rights, and the commodification of housing drove up urban property prices—further widening the urban-rural wealth gap.

Furthermore, the housing reform and the hukou system have jointly exacerbated urban-rural inequality, thereby entrenching disparities in wealth accumulation. Urban housing has become a tradable asset, creating a wealth cycle driven by price appreciation. In contrast, rural homesteads are subject to restrictions and difficult to capitalize. The hukou system excludes rural migrants from urban housing benefits and asset accumulation, forcing their savings into low-value rural homes. As a result, the urban-rural gap has shifted from a residential gap to an asset gap, making it a core driver of China’s widening wealth inequality.

Second, narrowing the urban-rural wealth gap is the core demand of common prosperity in Chinese society. Economically defined, “prosperity” refers to the possession of substantial money, material goods, housing, land, and other assets, while “common” means that the general population can ultimately achieve such prosperity. The pursuit of common prosperity is a process in which social wealth continues to accumulate, and wealth differences between urban and rural areas, as well as across regions, gradually diminish.

To formulate phased development goals for common prosperity, it is necessary to reasonably measure China’s current level of wealth inequality. Among various inequality metrics, the Gini coefficient is one of the most widely accepted. By defining the between-group Gini coefficient, Dagum proposed a complete decomposition of the Gini coefficient by group [5], enabling researchers to calculate the urban-rural Gini coefficient as the between-group Gini coefficient between urban and rural populations. For measuring wealth inequality, Piketty et al. and the WID prefer three core indicators to visually describe it: the wealth shares of the bottom 50%, top 10%, and top 1% of the population [6–8]. The wealth share of the top 10% reflects the wealth level and concentration among the richest group, while the wealth share of the bottom 50% indicates the wealth status of the poorest group. A higher wealth share of the top 10% and a lower wealth share of the bottom 50% indicate a wider wealth gap between the two groups.

To facilitate the analysis of the structure of wealth inequality, this paper uses the wealth Gini coefficient. There are two main methods to estimate the wealth Gini coefficient: on the one hand, statistical indicators such as decile data can be used [9–14]. Based on the wealth shares of the bottom 40% and top 10% [15,16], Dai and Shen provided a valid estimate of the wealth Gini coefficient [17]. On the other hand, the wealth Gini coefficient can be directly calculated using household survey data. However, Gini coefficients derived from household surveys are often underestimated because high-net-worth households are reluctant to participate, leading to their underrepresentation. To address the low participation rate of high-net-worth households in surveys, some scholars have proposed methods that combine rich list data with household survey data to adjust Gini coefficient estimates and tackle related issues such as distributional correction, tail modeling, reweighting, and top-share measurement. [6,18–26].

Among various correction methods, the generalized Pareto interpolation method proposed by Blanchet et al. [19] performs well in estimating the wealth Gini coefficient. Additionally, robust software support is available on the WID.world website, greatly facilitating applied research on wealth Gini coefficient correction. Therefore, this paper adopts the generalized Pareto interpolation method to adjust China’s wealth Gini coefficient.

Based on data from the China Family Panel Studies (CFPS) 2012–2022 and the Hurun Rich List, this paper corrects the urban-rural wealth Gini coefficient by aligning with the WID’s estimates of China’s overall wealth Gini coefficient. It also reveals the characteristics of wealth inequality in China under the hukou system, which constitutes the Chinese segment of the global wealth distribution. This paper makes four main contributions.

First, household net assets are decomposed into household assets and household liabilities. Household assets include land, real estate, financial assets, productive fixed assets, and durable consumer goods, while household liabilities are divided into mortgage loans and non-mortgage loans. This decomposition reveals the structural characteristics of Chinese household wealth: real estate and financial assets are the most important components. Real estate accounts for more than 75% of household net assets, and financial assets account for more than 10%. Meanwhile, real estate inequality explains over 75% of household wealth inequality, and financial asset inequality accounts for nearly 10% of household wealth inequality.

Second, this paper proposes a between-group Gini coefficient algorithm suitable for household data, which decomposes the overall wealth Gini coefficient into within-group and between-group wealth inequality across urban and rural areas. The results show that urban-rural wealth inequality accounts for more than 50% of the overall wealth inequality, indicating that reducing urban-rural wealth inequality is crucial to narrowing the overall wealth gap.

Third, this paper employs the generalized Pareto distribution and incorporates rich list data to adjust the urban wealth Gini coefficient, the urban-rural wealth Gini coefficient, and the overall wealth Gini coefficient. This approach addresses the underestimation of the wealth Gini coefficient caused by the underrepresentation of high-net-worth households in surveys.

Fourth, this paper provides a specific method for reasonably calculating China’s urban-rural wealth Gini coefficient. This method is based on household survey data, combined with rich list data and the WID’s estimates of China’s overall wealth Gini coefficient.

The remainder of this paper is structured as follows: Section 2 presents the materials and methods, Section 3 includes the results and discussion, and Section 4 documents the conclusions.

## 2. Materials and methods

### 2.1. Ethical approval

Ethical approval was not required as the study did not involve human participants.

### 2.2. Data materials

#### 2.2.1. CFPS2012–2022.

The China Family Panel Studies (CFPS) has published seven times to date, producing the databases CFPS2010, CFPS2012, CFPS2014, CFPS2016, CFPS2018, CFPS2020, and CFPS2022. The survey includes household income, consumption, wealth, and basic demographic information, and provides great convenience for many inequality studies of government and public concern. Piketty et al. use information from Chinese household surveys, including CFPS, in their study of wealth inequality in China [6].

In CFPS, household net assets are equal to total household assets minus total household liabilities. Household assets include land, real estate, financial assets, productive fixed assets, and durable consumer goods. Financial assets include deposits, stocks, funds, bonds, financial derivatives, other financial products, and loans. Productive fixed assets include business enterprise assets and agricultural machinery, among others. Durable consumer goods include common household items such as cars, televisions, computers, and refrigerators. Household liabilities include housing liabilities and non-housing liabilities. Housing liabilities are derived from the respondents’ self-reported ’mortgages with principal and interest not yet paid off’ during the survey. Non-housing liabilities come from debts related to education, medical care, and other areas.

Considering that the 2010 survey does not include government bonds, financial derivatives and other financial products, and is different from the asset caliber of other years, in order to ensure that the wealth data of each year are comparable, this paper only uses the survey data of the following six years. At the same time, according to the purpose of the study, only complete records of net asset value and urban-rural classification information are retained for estimating the Gini coefficient of overall, urban, rural and urban-rural wealth. The net assets as wealth and related information are shown in Table 1, with a marked increase over time in the wealth levels of both urban and rural residents. In the past 10 years, the per capita wealth increase of urban residents is significantly greater than that of rural residents [27], Without considering factors such as inflation, according to CFPS 2012–2022, the per capita wealth of urban residents increased from 150,734 CNY in 2012–392,112 CNY in 2022, a rise of 241,378 CNY, representing a growth rate of 160%. The per capita wealth of rural residents increased from 47,077 CNY in 2012–109,249 CNY in 2022, a rise of 62,172 CNY, representing a growth rate of 132%. Over this ten-year period, both the absolute increase and the growth rate of wealth were higher for urban residents than for rural residents.

**Table 1:** Basic information of CFPS2012-2022.

Year	family	Urban			Rural			Household wealth		STD.
		Family	Person	Wealth	family	Person	Wealth	Min.	Max.	
				per capita			per capita			
2012	12798	5872	20595	150733.95	6926	28566	47076.71	−1959200	34084688	778130.43
2014	13041	6377	21852	180403.49	6664	27084	57623.28	−2255000	18528900	842175.37
2016	13247	6640	22700	238158.31	6607	26652	66936.11	−79916375	80130000	1737419.88
2018	13226	6798	22807	347188.16	6428	24949	93924.19	−2470000	50461000	1851076.51
2020	10196	5499	18708	329154.31	4697	18732	92135.82	−9710250	61800000	1925574.58
2022	9571	5261	17242	392111.53	4310	16293	109249.16	−10040000	40975000	2221040.41

^1^ **Family unit is per household, person unit is per person; wealth unit is CNY.**

^2^ **Min., Max., and STD. represent the minimum, maximum, and standard deviation of the household asset from survey, respectively.**

#### 2.2.2. The Hurun rich list.

Since 2000, Hurun has released the Rich List of China mainland year by year. The number of people on the list has grown from 50 in 2000 to more than 1000 later. The use of the Rich List is mainly to compensate for the lower participation rate of high-net-worth groups in household surveys [6,18–26]. By estimating the wealth quantile of the high-end population, the generalized Pareto interpolation method can be used to estimate the wealth Gini coefficient based on the data from CFPS [19]. The basic information of the Rich List in the corresponding year is listed in Table 2, and it is clear that the threshold for inclusion in the list has gradually increased with economic growth.

**Table 2:** The Huren Rich List and population of China.

Year	Person	Total	Wealth	Population		Quantile Point (%)		Urbanization
		wealth	threshold	Urban	Rural	Total	Urban	Rate
2012	1024	55130	18	71182	64222	99.99962	99.99928	52.57
2014	1271	81539	20	74916	61866	99.99954	99.99915	54.77
2016	1400	131985	30	79298	58973	99.99949	99.99912	57.35
2018	1400	157432	30	86433	54108	99.99950	99.99919	61.50
2020	1400	246230	40	90220	50992	99.99950	99.99922	63.89
2022	1305	244769	50	92071	49104	99.99954	99.99929	65.22

^1^ **The number of people on the list, the wealth threshold for being listed, and the total wealth data are calculated based on the Hurun China Rich List. person unit is per person, wealth unit is 100 million CNY.**

^2^ **The population data of urban and rural areas comes from National Bureau of Statistics of China. Population unit is ten thousand.**

^3^ **A quantile point is equal to 1 minus the population share of the Huren Rich List.**

^4^ **The urbanization rate refers to the proportion of the urban population at the end of the year, with data sourced from the National Bureau of Statistics of China.**

A quantile point refers to the cumulative population share up to the last on the Hurun Rich List, which is equal to 1 minus the population share of the list. For example, in 2012, there are 1,024 people on the list. Following the approach of Piketty et al. [6], each listed person corresponds to a household of five adults, so the list covers 5,120 people. The population at the end of 2012 was 1,354.04 million, resulting in a list population share of 0.0003781%, corresponding to a quantile point of 99.99962% for nation. In addition, using the Forbes Rich List to obtain the wealth distribution of high-net-worth individuals is also an option [28].

#### 2.2.3. The WID’s estimates of wealth Gini coefficient of China.

WID is an international academic institution initiated by internationally renowned scholars such as Piketty et al., which regularly provides inequality indicators such as wealth shares of the bottom 50%, top 10%, top 1% of the population and Gini coefficient in each economy. Using information such as China’s macro account, CFPS and the Hurun Rich List, WID estimates wealth Gini coefficients of China over the years. Adopting the approach of Piketty et al. [6], the wealth Gini coefficient of China in WID can be restored, and the urban-rural wealth Gini coefficients can be adjusted by the obtained adjustment factor.

**Fig 1:**
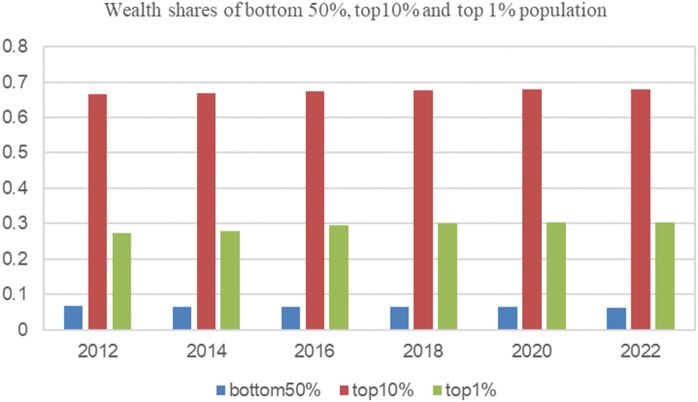
Shares of wealth owned by the top 1%, top 10%, and bottom 50% of the population in China (2012-2022). Data sources: World Inequality Database, https://wid.world.

Fig 1 presents the shares of wealth owned by the top 1%, top 10%, and bottom 50% of the population in China, as estimated by WID based on China data such as CFPS2012–2022. In 2018, 2020, and 2022, the top 1% held more than 30% of the wealth, while the bottom 50% held less than 7%, indicating that missing wealth records of the top population would significantly impact the wealth distribution.

### 2.3. Measurement of urban-rural wealth inequality

#### 2.3.1. Measuring Gini coefficient between groups.

There are *n* individuals, which are arranged in ascending order according to individual wealth as $y_{1}\leq y_{2}\leq \ldots \leq y_{n}$, Dividing these individuals into *k* groups is equivalent to splitting the subscript set N=1,2,…,n into *k* subsets of subscripts $N=N_{1}+N_{2}+\ldots +N_{k}$. The number of individuals in each subset is denoted as $n_{1},n_{2},\ldots ,n_{k}$, Dagum gives a definition of the Gini coefficient between groups *j* and *h* [5]:


$$G_{jh}=\frac{1}{{\bar{y}}_{j}+{\bar{y}}_{h}}\sum_{i\in N_{j}}\sum_{r\in N_{h}}\left|y_{i}-y_{r}\right|\frac{1}{n^{2}},\;{\bar{y}}_{j}=\frac{1}{n^{j}}\sum_{i\in N_{j}}y_{i},\;j,h=1,2,\ldots ,k$$ (1)

Eq (1) can be extended to the form of grouped data:

There are *n* families, arranged in order of increasing per capita wealth as $y_{1}\leq y_{2}\leq \ldots \leq y_{n}$; the number of person in each family is $q_{1},q_{2},\ldots ,q_{n}$, respectively, and the total population is *q*, which is obviously q≥n. If *n* families are still divided into group *k*, and the population of each group is expressed as $q^{1},q^{2},\ldots ,q^{k}$ by superscript, then the Gini coefficient between groups *j* and *h* can be defined as:


$$G_{jh}=\frac{1}{{\bar{y}}_{j}+{\bar{y}}_{h}}\sum_{i\in N_{j}}\sum_{r\in N_{h}}\left|y_{i}-y_{r}\right|\frac{q_{i}}{q^{j}}\frac{q_{r}}{q^{h}},\;{\bar{y}}_{j}=\frac{1}{q^{j}}\sum_{i\in N_{j}}q_{i}y_{i},\;j,h=1,2,\ldots ,k$$ (2)

When *j* = *h*, the between-group Gini coefficient becomes the within-group Gini coefficient.

#### 2.3.2. Measuring the urban-rural Gini coefficient.

The *n* families are divided into two groups according to urban and rural types, and *j* = 1 and *h* = 2 may be set to represent urban group and rural group respectively. Eq (2) can be deformed as:


$$G_{12}=\frac{1}{{\bar{y}}_{1}+{\bar{y}}_{2}}\sum_{i\in N_{1}}\sum_{r\in N_{2}}\left|y_{i}-y_{r}\right|\frac{q_{i}}{q^{1}}\frac{q_{r}}{q^{2}}$$ (3)

Eq (3) represents the between-group Gini coefficient for urban group and rural group (referred to as the urban-rural Gini coefficient).

As within-group Gini coefficient, total, urban, and rural Gini coefficients can be expressed as:


$$G=\frac{1}{2\bar{y}}\sum_{i\in N}\sum_{r\in N}\left|y_{i}-y_{r}\right|\frac{q_{i}}{q}\frac{q_{r}}{q},\;G_{kk}=\frac{1}{2{\bar{y}}_{k}}\sum_{i\in N_{k}}\sum_{r\in N_{k}}\left|y_{i}-y_{r}\right|\frac{q_{i}}{q^{k}}\frac{q_{r}}{q^{k}},\;k=1,2$$ (4)

This is the Gini mean deviation definition of the Gini coefficient, and household wealth can be negative, requiring only total wealth to be greater than 0. Therefore, the range of the wealth Gini coefficient is any positive real number, and the Gini coefficient is equal to 0 when everyone has the same wealth [14,29].

Regarding negative net assets, typical practices include deleting negative observations, replacing negatives with zero [30], standardizing the Gini coefficient with dynamic adjustment factors [31], or constructing nonnegative distributions by translating extremes [32]. However, these fixes systematically underestimate true inequality [29]. Moreover, because they depend on sample-specific choices, differences in exclusion rates or normalization factors across samples can introduce bias in cross-period or cross-regional comparisons.

As an example, we take the Gini coefficient measuring the wealth gap between two provinces to illustrate the between-group Gini coefficient: CFPS2022 has 9,929 valid wealth records (households), including 134 from Beijing and 476 from Shanghai. The wealth gap between residents in Beijing (Group A) and Shanghai (Group B) can be expressed using the between-group Gini coefficient. Taking one household from the 134 households in Group A and one household from the 476 households in Group B, calculating the difference in per capita wealth for each pair, and then calculating the weighted average of the absolute values of these differences (there are 63,784 combinations), the between-group Gini coefficient is obtained as 0.6434. In other words, the Gini coefficient between groups A and B is the weighted average of the per capita wealth differences between any two households from Beijing and Shanghai in the CFPS2022.

The within-group Gini coefficient for Beijing (Group A) is calculated by taking per capita wealth value from each of the 134 families twice (with possible repeats), calculating the difference between two values, and then taking the weighted average of the absolute values of the differences, the within-group Gini coefficient is obtained as 0.6963. In essence, it is the Gini mean difference of the wealth of the 134 families divided by twice the per capita wealth of all families.

### 2.4. Decomposition of the wealth Gini coefficient

#### 2.4.1. Decomposition of the wealth Gini coefficient by sources.

The decomposition of the wealth Gini coefficient by sources breaks the Gini coefficient down into the sum of concentration indices based on r sources of wealth:


$$G=s_{1}C_{1}+s_{2}C_{2}+\ldots +s_{r}C_{r}$$ (5)

where *C*_*i*_ represents the concentration index of the *i*th source of wealth, and *s*_*i*_ represents the share of wealth of the *i*th source (i=1,2,…,r). Eq (5) is similar to the decomposition form suggested by Lerman and Yitzhaki [33].

#### 2.4.2. Decomposition of the wealth Gini coefficient by groups.

Decomposing the total Gini coefficient into urban and rural groups, we can obtain a complete decomposition of the Gini coefficient:


$$G=\sum_{i=1}^{2}s_{i}p_{i}G_{ii}+(s_{1}p_{2}+s_{2}p_{1})G_{12}$$ (6)

where $s_{1},s_{2},p_{1}$ and *p*_2_ represent shares of wealth and population of urban and rural groups, respectively. The first two terms on the right side of Eq (6) are collectively called within-group inequality of the Gini coefficient, and the third term is called between-group inequalities. The contribution of within- and between-group inequalities to the total Gini coefficient can be calculated separately.

### 2.5. Corrective methods for the missing high-net-worth population

Blanchet et al. [19] proposed a method to estimate wealth quantiles and Gini coefficients by fitting a generalized Pareto distribution using several wealth quantiles and the mean wealth of the right interval. The absolute error between the wealth Gini coefficient estimated by this method and the sample Gini coefficient is within 0.5%. The method usually requires 10 statistics: the wealth threshold corresponding to the 4 wealth quantiles and the mean wealth on their right-side interval (e.g., the bottom 10%, 50%, 90%, and 99% quantiles), plus the population size and the total mean wealth. WID provides the calculation software ‘gpinter’ on its website WID.world to help researchers process the data and obtain estimates of wealth quantiles and wealth Gini coefficients.

### 2.6. Validity of the Gini coefficient estimates

The Gini coefficient is usually expressed using two pure decimal places, and the third decimal place is rounded. Therefore, similar to the practice of Dai and Shen [17], it is reasonable to control the absolute deviation between the true and estimated values of Gini coefficient in the thousandth percentile. When the deviation is kept to two significant figures, the absolute deviation of the estimated value from the true value is less than or equal to 0.01 after rounding from the thousandth place, and the estimate is said to be a valid estimate of the Gini coefficient. It is equivalent to:

**Definition 1** If the absolute deviation of the estimated Gini coefficient from the true Gini coefficient is less than 0.015, then the estimate is called a valid estimate of the Gini coefficient.

For two valid estimates of the same Gini coefficient, we require the absolute difference between the two to be less than 0.015.

## 3. Results and discussion

### 3.1. The urban-rural wealth Gini coefficient

#### 3.1.1. Differences in the wealth between urban group and rural group.

Table 3 reports the structure of CFPS assets and liabilities for each year. Overall, the wealth structure is relatively obvious and stable: real estate is the most important asset of residents, accounting for more than 75%; then there are financial assets, which account for more than 10% and show an increasing trend. From the perspective of the asset composition of urban and rural residents, the proportion of real estate is significantly higher for urban residents, exceeding 80%, while the proportion of durable consumer goods associated with real estate is higher for rural residents. The second largest asset owned by urban residents is financial assets, while the second largest asset owned by rural residents is land assets, which manifests in the fact that more of the consumption surplus of urban residents is used for saving and purchasing financial products, and land is the basic condition of rural residents for their survival. Due to the needs of production and operation, the proportion of non-housing loan of rural residents is higher than that of urban residents, and the proportion of fixed assets of rural residents is higher than that of urban residents.

**Table 3:** Structure of assets and liabilities of CFPS (%).

Types	Year	Financial	Fixed	Land	Consumer	Real	Housing	Other
		assets	assets	assets	durable	estate	loans	loans
**Urban**	2012	10.76	5.32	2.29	5.02	81.15	−2.02	−2.53
	2014	11.04	2.98	1.56	5.26	85.03	−4.10	−1.78
	2016	11.91	4.57	1.34	5.94	83.34	−5.41	−1.69
	2018	10.12	3.26	0.98	5.25	86.75	−4.91	−1.45
	2020	14.51	4.01	1.14	6.56	83.81	−8.13	−1.90
	2022	15.94	3.22	0.88	6.91	83.82	−8.29	−2.48
**Rural**	2012	9.40	9.43	19.84	5.42	63.30	−1.81	−5.57
	2014	8.65	4.62	16.44	6.16	74.22	−5.76	−4.32
	2016	10.57	6.67	13.50	8.14	76.16	−10.63	−4.42
	2018	10.22	6.13	8.44	7.45	78.14	−6.28	−4.10
	2020	13.85	5.13	10.77	9.19	74.89	−9.45	−4.37
	2022	15.26	5.88	10.30	9.84	72.62	−9.34	−4.57
**Total**	2012	10.35	6.56	7.60	5.14	75.76	−1.95	−3.45
	2014	10.36	3.45	5.78	5.52	81.96	−4.57	−2.50
	2016	11.58	5.10	4.36	6.48	81.56	−6.71	−2.37
	2018	10.14	3.92	2.68	5.75	84.78	−5.22	−2.05
	2020	14.36	4.26	3.25	7.13	81.86	−8.41	−2.44
	2022	15.80	3.78	2.84	7.52	81.49	−8.51	−2.91

^1^Liabilities are divided into housing loans and other loans. Productive fixed assets are simply referred to as fixed assets.

^2^The ratios of various assets and liabilities are calculated using CFPS data.

#### 3.1.2. Decomposition of the wealth Gini coefficient.

Decomposition of the wealth Gini coefficient by sources. Table 4 reports the concentration index of various types of assets of CFPS, indicating that there are large differences in the accumulation of fixed assets, real estate and financial assets. Multiplying the concentration index of each asset by the share of each asset can calculate their unequal contribution to the wealth Gini coefficient, the results of which are presented in Table 5.

**Table 4:** Asset and liability concentration index of CFPS2012-2022.

Year	Financial	Fixed	Land	Consumer	Real	Housing	Other
	assets	assets	assets	durable	estate	loans	loans
2012	0.6351	0.8278	0.2452	0.5481	0.6873	0.5769	−0.0179
2014	0.6495	0.6181	0.2088	0.5082	0.6831	0.3086	−0.1937
2016	0.6601	0.7347	0.2327	0.5447	0.7142	0.1357	−0.1605
2018	0.6623	0.7668	0.1513	0.5389	0.7110	0.4596	0.0193
2020	0.6757	0.7586	0.2425	0.5320	0.7127	0.4308	−0.0247
2022	0.6668	0.7266	0.2219	0.5182	0.7130	0.4298	−0.1155

^1^ **The concentration index of various assets and liabilities is calculated using CFPS data.**

**Table 5:** Inequality contribution rate of assets and liabilities of CFPS (%).

Year	Financial	Fixed	Land	Consumer	Real	Housing	Other
	assets	assets	assets	durable	estate	loans	loans
2012	9.71	8.03	2.75	4.16	76.93	−1.67	0.09
2014	9.91	3.14	1.78	4.13	82.42	−2.08	0.71
2016	10.38	5.08	1.38	4.80	79.08	−1.24	0.52
2018	9.45	4.23	0.57	4.36	84.82	−3.38	−0.06
2020	13.43	4.47	1.09	5.25	80.70	−5.02	0.08
2022	14.51	3.78	0.87	5.37	80.04	−5.04	0.46

^1^ **The concentration index of various assets and liabilities is calculated using CFPS data.**

Table 5 illustrates that real estate contributes the most to the wealth Gini coefficient among the various types of asset inequality, followed by financial assets.

Decomposition of the wealth Gini coefficient by groups. Table 6 reports the results of the wealth Gini coefficient and the decomposition of inequality for CFPS. The share of urban population in the sample is much lower than the real level of national urbanization (see Tables 3 and 6), but with the increase of urban population share, the share of urban wealth increases almost simultaneously, and the wealth share is nearly 30% higher than population share. From the perspective of wealth inequality, the contribution of rural inequality, urban inequality and urban-rural inequality increases in turn, and the contribution rate of between-group inequality is significantly greater than that of within-group inequality. The contribution rate of between-group inequality to the overall wealth Gini coefficient is more than 50%, indicating that the urban-rural wealth gap is the main factor causing wealth inequality in China.

**Table 6:** The wealth Gini coefficient, the sample urbanization rate and the decomposition of inequality (%).

Year	Sample Gini coefficient				Urban		Decomposition			Contribution rate	
	Total	Urban	Rural	Urban-	Population	Wealth	Urban	Rural	Urban-	Within	Between
				rural	share	share			rural	group	group
2012	0.6768	0.6548	0.5857	0.7190	41.89	69.77	0.1914	0.1029	0.3826	43.48	56.52
2014	0.6793	0.6610	0.5910	0.7171	44.65	71.64	0.2115	0.0928	0.3751	44.78	55.22
2016	0.7365	0.6996	0.7004	0.7704	46.00	75.19	0.2419	0.0938	0.4007	45.59	54.41
2018	0.7106	0.6637	0.6507	0.7584	47.76	77.16	0.2446	0.0776	0.3884	45.34	54.66
2020	0.7229	0.6955	0.6413	0.7620	49.97	78.11	0.2714	0.0702	0.3811	47.27	52.72
2022	0.7259	0.7003	0.6392	0.7649	51.41	79.16	0.2850	0.0647	0.3761	48.18	51.82

^1^ **Each indicator is calculated based on CFPS data.**

**Fig 2:**
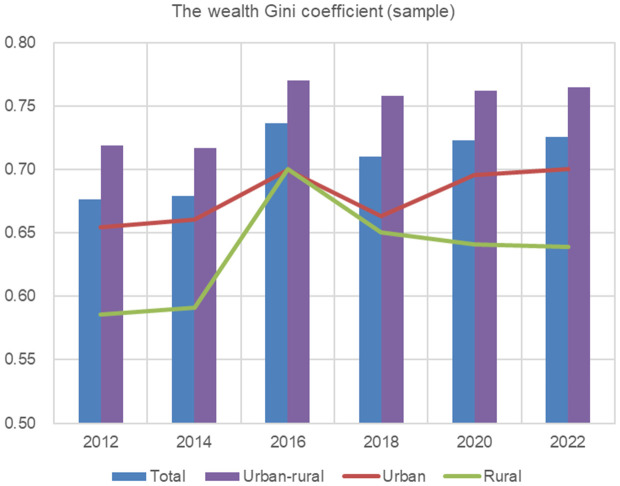
Overall, urban, rural and urban-rural wealth Gini coefficients (CFPS2012-2022). Data sources: China Family Panel Studies, https://cfpsdata.pku.edu.cn.

Based on the calculation results of the sample Gini coefficient for the CFPS 2012–2022 in Table 6, Fig 2 shows the trends of the total, urban, rural, and urban-rural wealth Gini coefficients. The total, urban, and urban-rural wealth Gini coefficients all show a certain degree of increase, while the rural wealth Gini coefficient exhibits an inverted U-shaped pattern, rising first and then falling.

**Fig 3:**
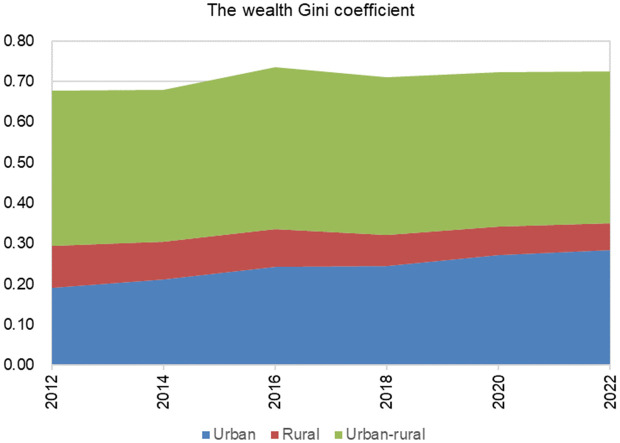
Decomposition of overall wealth inequality (2012-2022). Data sources: China Family Panel Studies, https://cfpsdata.pku.edu.cn.

Fig 3 shows the visual composition of the three sources of inequality in the total wealth Gini coefficient. Urban-rural inequality is the main source of wealth inequality, followed by urban wealth inequality. As China urbanizes, the contribution of rural wealth inequality to overall wealth inequality has gradually shrunk. The contribution of urban wealth inequality is increasing year by year. It can be seen that in order to achieve common prosperity, narrowing the urban-rural wealth gap is the key, and at the same time, measures should be taken to control the rising trend of urban wealth inequality.

### 3.2. Correction of the urban-rural wealth Gini coefficient

From the decomposition of the wealth Gini coefficient in Eq (6), the formula for calculating the urban-rural wealth Gini coefficient can be obtained:


$$G_{12}=\frac{G-s_{1}p_{1}G_{11}-s_{2}p_{2}G_{22}}{s_{1}p_{2}+s_{2}p_{1}}$$ (7)

Considering that the individuals who can enter the Hurun Rich List are usually urban residents [34], stitching data from the Rich List to correct the overall wealth Gini coefficient only has an impact on the urban wealth data, so the Gini coefficient of rural wealth remains unchanged. Therefore, the basic way to correct the urban-rural wealth Gini coefficient is to restore the WID estimate of the wealth Gini coefficient of China in the corresponding year, and the urban wealth Gini coefficient can be corrected at the same time. The corrected value of the urban-rural wealth Gini coefficient is obtained from the adjusted overall wealth Gini coefficient, the adjusted urban wealth Gini coefficient, and the unadjusted rural wealth Gini coefficient, using Eq (7).

#### 3.2.1. Correction of the overall wealth Gini coefficient.

Table 7 based on data from CFPS2012, reports how to reconstruct WID’s estimate of the wealth Gini coefficient of China in conjunction with the Hurun Rich List. It can be obtained by combining the CFPS2012 with the 2012 Hurun Rich List:

**Table 7:** Correction of the overall wealth Gini coefficient by the generalized Pareto interpolation.

Quantile point	Wealth threshold	The mean wealth of the right-side interval	Adjustment factor	Adjusted mean wealth
10%	6675	20204.18	—	20204.18
50%	36125	79631.62	—	79631.62
90%	192475	506180.08	1.4	708652.12
99.9996%	360000000	1076757813	—	1076757813
**The total population of China mainland at the end of 2012**				1354040000
**National mean wealth**				114765.90
**The total wealth Gini coefficient before interpolation**				0.6768
**The total wealth Gini coefficient after interpolation**				0.7452

^1^ **The adjustment factor 1.4 for the mean wealth of the top 10% is obtained based on the estimated wealth Gini coefficient of 0.7477 from WID. Wealth unit is CNY. We use the trial-and-error method, adjusting the factor successively to 1.3, 1.4, and 1.5 for testing. After inserting the data of Hurun Rich List, the total wealth Gini coefficients are 0.7342, 0.7452, and 0.7553, respectively, which deviate from the WID estimate of 0.7477 by 0.0135, 0.0025, and −0.0076. Therefore, we chose 1.4 as the adjustment factor, as it resulted in the smallest absolute deviation.**

^2^ **The national mean wealth is equal to the weighted average of the mean wealth of each interval after adjusting the splice interval mean wealth, the mean wealth for the interval (0%,10%) −614 is obtained from CFPS2012.**

The quantile data of the 2012 Rich List are inserted to convert the sample data into national data in Table 7. The wealth threshold of the list is equal to 360000000, which is higher than the upper limit of the CFPS2012 as 34084688 (see Tables 1 and 2), and the mean wealth of the right-side interval is equal to 1076757813. Here, it is assumed that each household on the list has 5 members [6].

For obtaining the mean wealth of the splice interval (90%, 99.9996%), we refer to the practice of Piketty et al. citebib6. Assuming that the wealth data of the bottom 90% of the survey in the CFPS2012 is credible, the mean wealth of the top 10% can be multiplied by an adjustment factor as the mean wealth of the splice interval. The 2012 wealth Gini coefficient of China given by WID is equal to 0.7477. In order to restore the value using data from the CFPS2012, several values of the adjustment factor are tried, and it is found that when the adjustment factor takes 1.4, the corrected wealth Gini coefficient is equal to 0.7452, and the absolute error of the two is less than 0.5%. Therefore, the mean wealth of the splice interval is equal to the mean wealth of the top 10% of the CFPS2012 times 1.4, that is 708652.12 CNY.

Due to the adjustment of the mean wealth of the splice interval, the national mean wealth should also be adjusted accordingly. The mean wealth of the right-side intervals of quantiles 0%, 10%, 50%, 90% and 99.9996% are weighted to obtain the national mean wealth of 114,765.9 CNY. Finally, the 10 statistics in Table 7 are stored in the CSV file in the format of WID.world, and the information such as the corrected wealth Gini coefficient can be obtained by running the ‘gpinter’ software.

The above process is the correction of the wealth Gini coefficient by combining the CFPS with the Rich List and using the generalized Pareto interpolation method.

#### 3.2.2. Correction of the urban wealth Gini coefficient.

The families on the Rich List belong to the urban population. In China, properties located in urban areas usually have higher value, as good living resources such as schools, hospitals, and supermarkets are concentrated in urban areas. Once rural residents’ economic conditions improve, they tend to purchase commercial housing in urban areas to facilitate their children’s education, employment, and improve the live conditions of families. Urbanization in China promotes the transformation of wealthy farmers into urban residents. For instance, a related study found that there are 1241 entrepreneurs entered the 2023 Hurun Rich List, they come from 144 cities [34].

Thus, similar to the processing of national data, the urban wealth Gini coefficient can be corrected. Table 8 shows the 10 statistics to correct the urban wealth Gini coefficient using the generalized Pareto interpolation method and the correction results of the urban wealth Gini coefficient, where the value of the adjustment factor is the same as that in Table 7.

**Table 8:** Correction of the urban wealth Gini coefficient (2012).

Quantile point	Wealth threshold	The mean wealth of the right-side interval	Adjustment factor	Adjusted mean wealth
10%	9419.44	34714.28	—	34714.28
50%	66000	147286.96	—	147286.96
90%	355058.33	780501.82	1.4	1092702.54
99.9993%	360000000	1076757813	—	1076757813
**The urban population of China mainland at the end of 2012**				711820000
**National urban mean wealth**				189528.70
**The urban wealth Gini coefficient before interpolation**				0.6548
**The urban wealth Gini coefficient after interpolation**				0.7247

^1^ **The adjustment factor 1.4 for the mean wealth of the top 10% is obtained based on the estimated wealth Gini coefficient of 0.7477 from WID.**

^2^ **The national average wealth is equal to the weighted average of the mean wealth of each interval after adjusting the splice interval mean wealth, the mean wealth for the interval (0%,10%) −1589.80 is obtained from CFPS2012.**

Based on the CFPS2012, the urban wealth Gini coefficient is equal to 0.6548, it is corrected to 0.7274 by combining with data from the Hurun Rich list, and the urban wealth Gini coefficient has changed greatly.

#### 3.2.3. Correction of the urban-rural wealth Gini coefficient.

Table 9 reports the corrected result of the urban-rural wealth Gini coefficient in 2012. It is obtained from Eq (7) based on correction to the total and urban wealth Gini coefficients, where the rural wealth Gini coefficient remains unchanged. The urban-rural wealth Gini coefficient rises from 0.7190 to 0.7921 after correction.

**Table 9:** Correction of the urban-rural wealth Gini coefficient using the generalized Pareto interpolation (2012).

Types	Sample	Adjustment	Population	Wealth	Corrected
Gini coefficient	factor	share	share	Gini coefficient
Total	0.6768	1.4	1	1	0.7452
Urban	0.6548	1.4	0.5257	0.8169	0.7247
Rural	0.5857	1	0.4743	0.1831	0.5857
Urban-rural	0.7190	—	—	—	0.7921

^1^ **Each indicator is calculated based on data from CFPS2012, the Hurun Rich List or WID.**

CFPS2014, CFPS2016, CFPS2018, CFPS2020, and CFPS2022 are processed in combination with data from the Hurun Rich List, and Table 10 reports the corrected results of the wealth Gini coefficients of total, urban, and urban-rural for the CFPS. The population share of urban is also the urbanization rate, and the wealth share of urban is calculated based on the wealth correction.

**Table 10:** The Gini coefficient and urban wealth share correction (2012-2022).

Year	The sample Gini coefficient				Adjusted Gini coefficient				Urban (adjusted)		
	Total	Urban	Rural	Urban-	WID	Total	Urban	Urban-	Adjustment	Population	Wealth
				rural				rural	factor	share	share
2012	0.6768	0.6548	0.5857	0.7190	0.7477	0.7452	0.7247	0.7921	1.4	52.57	81.69
2014	0.6793	0.6610	0.5910	0.7171	0.7492	0.7494	0.7318	0.7927	1.4	54.77	82.77
2016	0.7365	0.6996	0.7004	0.7704	0.7546	0.7573	0.7233	0.8023	1.0	57.35	83.64
2018	0.7106	0.6637	0.6507	0.7584	0.7558	0.7581	0.6991	0.8471	1.1	61.50	86.74
2020	0.7229	0.6955	0.6413	0.7620	0.7567	0.7610	0.7343	0.8121	1.1	63.89	87.80
2022	0.7259	0.7003	0.6392	0.7649	0.7574	0.7609	0.7363	0.8107	1.1	65.22	88.32

^1^ **Each indicator is calculated based on data from CFPS, the Hurun Rich List, WID and National Bureau of Statistics of China.**

^2^ **Adjustment factors for the mean wealth of the top 10% is obtained based on the estimated wealth Gini coefficients from WID.**

^3^ **Using the population data of urban and rural areas from Table 2, the adjusted wealth per capita of urban residents, and the sample wealth per capita of rural residents, the adjusted wealth share of urban residents can be calculated.**

**Fig 4:**
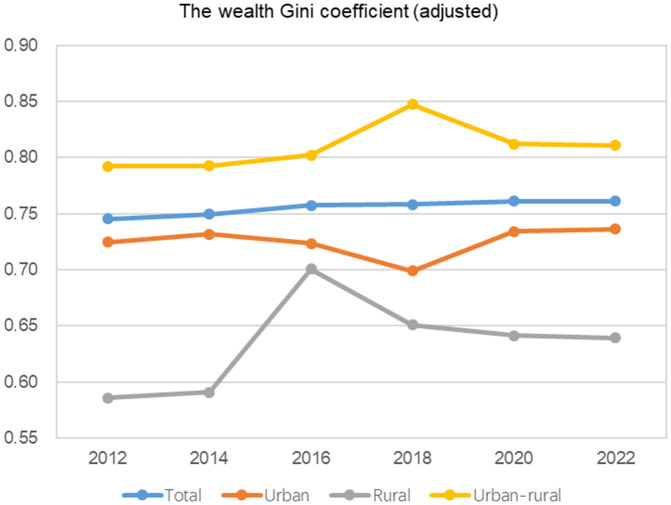
Overall, urban, rural and urban-rural wealth Gini coefficients of China (2012-2022). Data sources: China Family Panel Studies, https://cfpsdata.pku.edu.cn.

The results in Table 10 show that with the gradual increase in the level of urbanization in China (the urbanization rate increased from 52.57% in 2012 to 65.22% in 2022, an increase of 12.65% in 10 years), the share of wealth of urban residents will inevitably increase (6.63% in 10 years). By 2022, the proportion of wealth of urban residents, who account for 65.22% of the population, will reach 88.32%, although the gap between the share of wealth and the share of the population has decreased from 29.12% in 2012 to 23.1% in 2022, it still cannot change the fact that wealth is skewed towards urban residents. After the correction of the Hurun Rich List, the overall wealth Gini coefficient showed a slow upward trend, and the urban-rural wealth Gini coefficient shows an inverted U-shaped change, but the upward trend was obvious (see Fig 4). The Gini coefficient of urban wealth still maintains an upward trend, indicating that in the short term, urbanization has played a very limited role in narrowing the wealth gap between urban and rural residents.

### 3.3. Discussion on the robustness of the method

#### 3.3.1. The threshold at which data is reliable.

In the previous adjustment of China’s wealth Gini coefficient, considering the missing wealth records of high-net-wealth population, we assumed that the wealth data of the bottom 90% of the CFPS population were reliable. Then, can the method used in this paper produce similar results for other cases with reliable data? Here we assume that we can only judge that the wealth data of the bottom 80% of the population in CFPS is reliable, using data from CFPS and the Hurun Rich List to adjust China’s wealth Gini coefficient. We change the quantile selection strategy of the generalized Pareto interpolation method, choosing the bottom 10%, 50%, 80% of CFPS2022, and the corresponding cumulative population share of the 2022 Hurun Rich List. Tables 11, 12 and 13, report the results of adjusting the overall, urban, and urban-rural wealth Gini coefficients, respectively.

**Table 11:** Correction of the overall wealth Gini coefficient (2022).

Quantile point	Wealth threshold	The mean wealth of the right-side interval	Adjustment factor	Adjusted mean wealth
10%	10800	44623.05	—	44623.05
50%	87083.67	157425.89	—	157425.89
80%	272500	956308.89	1.05	1004124.3
99.9995%	1000000000	3751249042.15	—	3751249042.15
**The total population of China mainland at the end of 2022**				1411750000
**National mean wealth**				281518.10
**The total wealth Gini coefficient before interpolation**				0.7259
**The total wealth Gini coefficient after interpolation**				0.7575

^1^ **The adjustment factor 1.05 for the mean wealth of the top 20% is obtained based on the estimated wealth Gini coefficient of 0.7574 from WID.**

^2^ **The national average wealth is equal to the weighted average of the mean wealth of each interval after adjusting the splice interval mean wealth, the mean wealth for the interval (0%,10%) −16892.38 is obtained from CFPS2022.**

**Table 12:** Correction of the urbanl wealth Gini coefficient (2022).

Quantile point	Wealth threshold	The mean wealth of the right-side interval	Adjustment factor	Adjusted mean wealth
10%	16140	74724.64	—	74724.64
50%	149562.5	263635.61	—	263635.61
80%	444816.70	1424968.95	1.05	1496217.40
99.9993%	1000000000	3751249042.15	—	3751249042.15
**The urban population of China mainland at the end of 2022**				920710000
**National urban mean wealth**				432438.70
**The urban wealth Gini coefficient before interpolation**				0.7003
**The urban wealth Gini coefficient after interpolation**				0.7364

^1^ **The adjustment factor 1.05 for the mean wealth of the top 20% is obtained based on the estimated wealth Gini coefficient of 0.7574 from WID.**

^2^ **The national average wealth is equal to the weighted average of the mean wealth of each interval after adjusting the splice interval mean wealth, the mean wealth for the interval (0%,10%) −21502.20 is obtained from CFPS2022.**

**Table 13:** Correction of the urban-rural wealth Gini coefficient using the generalized Pareto interpolation (2022).

Types	Sample	Adjustment	Population	Wealth	Corrected
	Gini coefficient	factor	share	share	Gini coefficient
Total	0.7259	1.05	1	1	0.7575
Urban	0.7003	1.05	0.6522	0.8813	0.7364
Rural	0.6392	1	0.3478	0.1187	0.6392
Urban-rural	0.7649	—	—	—	0.8018

^1^ **Each indicator is calculated based on data from CFPS2022, the Hurun Rich List or WID.**

Choosing the third quantile point at 80% or 90% has little impact on the adjustments of the overall, urban, and urban-rural wealth Gini coefficients. In Table 13, choosing 80% as the quantile point yields the corrected Gini coefficients of overall, urban, and urban-rural wealth in 2022 as 0.7575, 0.7364, and 0.8018, respectively, with the corrected urban wealth share being 0.8813. Comparing with the corresponding 2022 adjusted values of 0.7609, 0.7363, 0.8107, and 0.8832 given in Table 10 for choosing 90% as the third quantile point, the respective absolute errors are 0.0034, 0.0001, 0.0089, and 0.0019, and their differences are all in the thousandths place or less. Their results are similar, mainly because the generalized Pareto interpolation method has favorable properties: first, the estimation error is in the thousandths; second, there are few restrictions on the choice of quantile points; third, it can measure subtle changes in the tails. Therefore, with changes in the threshold at which data is reliable, the adjustment of the wealth Gini coefficient is robust.

#### 3.3.2. The Forbes rich list.

In recent years, some scholars believe that the ranking rules of the Rich List are not transparent [19], and they are concerned that using the Rich List to adjust the wealth Gini coefficient carries certain risks. Next, we will try using the Forbes Rich List instead of the Hurun Rich List to observe the impact of different billionaire lists on the adjusted wealth Gini coefficient. We still use the data from 2022 as an example. We extracted the records of Chinese billionaires from the 2022 Forbes Global Billionaires List. The wealth threshold to make the list is 1 billion US dollars. There are 539 people from China Mainland on the list, with a total wealth of 1,962.45 billion US dollars. To facilitate data stitching between Forbes Rich List and CFPS, we convert 1 US dollar to RMB 6.7261 (average annual exchange rate in 2022). Therefore, the per capita wealth threshold for households on the list is 1,345,220,000 CNY, the per capita wealth is 4,897,823,727 CNY, and the corresponding overall cumulative population share is 99.9998%. WID estimates China’s wealth Gini coefficient at 0.7574 for 2022, from which an adjustment factor of 1.15 can be derived. Finally, based on the Forbes Rich List, the adjusted values of the overall, urban, and urban-rural wealth Gini coefficients for 2022 were 0.7598, 0.7347, and 0.8103, respectively, and the adjusted urban wealth share was 0.8830. Comparing with the corresponding adjusted values for 2022 given in Table 10 using the Hurun Rich List, which are 0.7609, 0.7363, 0.8107, and 0.8832, the absolute errors are 0.0011, 0.0016, 0.0004, and 0.0002, respectively, all differences being in the thousandths or less. Table 14 reports the results of adjusted China’s wealth Gini coefficient using CFPS and the Forbes Rich List data from other years. It can be seen that different rich lists have very limited impact on the adjustment of China’s wealth Gini coefficient.

**Table 14:** Correction of China’s wealth Gini coefficient Based on Forbes Rich List and CFPS2012-2022.

Year	Person	Total	Wealth	Adjusted Gini coeficient			Urban (adjusted, %)		
		wealth	threshold	Total	Urban	Rural	Adjusted	Population	Wealth
							factor	share	share
2012	400	26603	30	0.7406	0.7188	0.7882	1.40	52.57	81.37
2014	400	41628	43	0.7444	0.7255	0.7886	1.40	54.77	82.43
2016	400	63453	67	0.7492	0.7135	0.7948	1.00	57.35	83.16
2018	400	72954	58	0.7501	0.6904	0.8387	1.10	61.50	86.41
2020	400	140547	103	0.7534	0.7258	0.8051	1.10	63.89	87.45
2022	539	131996	67	0.7598	0.7347	0.8103	1.15	65.22	88.29

^1^ **The number of people on the list, the wealth threshold for being listed, and the total wealth data are calculated based on the Forbes China Rich List. person unit is per person, wealth unit is 100 million CNY.**

^2^ **The population share refers to the proportion of the urban population at the end of the year, with data sourced from the National Bureau of Statistics of China.**

^3^ **Wealth share refers to the proportion of wealth of urban residents after adjustment**

## 4. Conclusions

This paper is based on data from CFPS and the Hurun Rich List, using the generalized Pareto interpolation method to restore the WID estimates of China’s wealth Gini coefficient and obtain the adjusted factor, while also correcting the urban-rural wealth Gini coefficients. The corrected urban-rural wealth Gini coefficient shows a more pronounced inverted U-shaped trend: from 2012 to 2022, China’s urban-rural wealth Gini coefficient first rises from 0.7921 in 2012 to a peak of 0.8471 in 2018, and then gradually decreases to 0.8107 in 2022. However, it is still in an upward phase. This indicates that urban residents have a significant advantage in per capita wealth, and the wealth gap between urban and rural residents may further widen. Changes in wealth inequality relate to the ultimate goal of common prosperity where the general public can collectively achieve prosperity.

Based on CFPS data, the study found that real estate plays a very important role in the wealth structure of Chinese residents, accounting for more than 75% of the total wealth. In the contribution of various types of asset inequality to the total wealth Gini coefficient, the contribution rate of real estate is the largest among all types of assets at more than 75%, followed by financial assets. The characteristics of real estate for urban residents are more obvious, accounting for more than 80% of the total wealth. Financial assets rank second in the wealth structure of urban residents, accounting for more than 10% of the total wealth. The wealth Gini coefficient is decomposed by urban and rural groups, with the contribution rate of urban-rural wealth inequality exceeding 50%, while the contribution rate of rural wealth inequality gradually declines to below 10%. The contribution rate of urban wealth inequality has gradually increased with the increase of China’s urbanization level, from nearly 30% in 2012 to 40% in 2022. Considering that the inequality between urban and rural areas contributes to over 50% of the overall wealth inequality, we believe that improving public services such as education, healthcare, and elderly care in rural areas can effectively narrow the wealth gap between urban and rural regions.
